# A case of probable non-familial early onset Alzheimer dementia in a Hispanic male

**DOI:** 10.3402/jchimp.v2i2.18600

**Published:** 2012-07-16

**Authors:** Corey Ephrussi, Richard Alweis

**Affiliations:** 1 Department of Medicine, The Reading Hospital and Medical Center, Reading, PA, USA; 2 Department of Medicine, The Jefferson Medical College, Philadelphia, PA, USA

**Keywords:** early onset Alzheimer's dementia, Hispanic, Presenilin-1

## Abstract

**Background:**

Early onset Alzheimer's type dementia (EOAD) is usually familial and associated with mutations in the Presenilin-1 (PSEN1), Presenilin-2 (PSEN2) or amyloid precursor protein (APP) genes. It is rarely reported in patients of Hispanic descent.

**Case report:**

A 49-year-old Hispanic male developed significant cognitive impairment over a 4-year period. PET scan showed diminished metabolic activity in the posterior parietal/temporal lobes. Genetic testing revealed the presence of a PSEN1 gene mutation.

**Conclusion:**

Disparities in health care may account for an under-recognition of EOAD in the Hispanic population. Clinicians should test for EOAD in all patients with appropriate symptomatology, regardless of ethnicity. Early recognition and enrollment in clinical trials is vital to enhancing our understanding of the natural history and treatment of this condition.

A 49-year-old Puerto Rican male with obstructive sleep apnea and anxiety presented with memory loss. Symptoms began 4 years earlier with work impairment, including filling out reports, completing routine tasks, and poor concentration. This progressed, and he had difficulty driving, causing multiple motor vehicle accidents, and forcing retirement from the police force. There was no tobacco, drug, or alcohol abuse or exposure to chemicals or toxins. Family history was significant only for dementia in his mother, diagnosed in her seventies, with no other dementia reported in any other relatives, including his seven older siblings. Physical examination was unremarkable. Folstein mini-mental status exam (MMSE) score was 25/30.

Blood work-up including B12, folate, TSH, ceruloplasmin, and homocysteine levels were normal. Titers for lyme, ANA, RPR, and HIV were negative. Brain MRI was unremarkable, and specifically showed no changes of vasculitis or Creutzfeldt–Jacob disease; therefore, lumbar puncture was not performed. However, brain PET CT scan showed diminished metabolic activity involving bilateral posterior parietal and temporal lobes with normal frontal lobe activity. Neuropsychological assessment (RBANS-A) showed significant cognitive impairment in attention (8th percentile), immediate memory (0.2nd percentile), delayed memory (5th percentile) and semantic fluency (5th percentile). Genetic testing revealed PSEN1 gene mutation (codon 206, nucleotide 617 with Transversion G > C). His symptoms rapidly progressed within the first 12 months after diagnosis, and Folstein MMSE deteriorated to 20/30. There was no response to donepezil or memantine and he failed a clinical trial drug. After another 12 months, his Folstein MMSE deteriorated to 10/30, he became non-verbal and sexually aggressive; he now resides in a locked dementia unit at a nursing facility.

## Discussion

Alzheimer's disease is a heterogeneous progressive neurodegenerative condition, classified based on age of onset as familial early onset (<60 years) and sporadic late onset (>60 years) (1). While memory impairment is the shared feature in early and late onset Alzheimer's disease, apraxia and visuospatial dysfunction are the most common presenting symptoms of EOAD (2). Autosomal dominant early onset Alzheimer's dementia (EOAD) usually results from a mutation in one of three genes, PSEN1, PSEN2, or APP, resulting in deposition of neurotoxic beta-A4 peptide in neural plaques (3). An autosomal recessive APP gene mutation (A673V) causes disease only in the homozygous state (4). Unfortunately, while there are postulated to be many autosomal recessive genes contributing to the development of Alzheimer's, the identified autosomal recessive mutations are not abundant. Early literature suggested that autosomal dominant mutations accounted for 30–50% of all EOAD cases and 10% of all Alzheimer's dementia cases, but recent literature has questioned this previous data, due to a greater awareness of likely autosomal recessive EOAD conditions (5, 6). There are a number of reports describing higher prevalence of late onset Alzheimer's disease in the Hispanic population (7, 8). Additionally, Athan et al. first reported a Gly–Ala substitution in codon 206 of the PSEN1 gene in 8 of 19 unrelated Caribbean Hispanic families with early onset familial AD (9). Similarly, PSEN1 and APP mutations have been found in other ethnic groups with early onset AD (10). Despite these findings, there is a paucity of studies in this population, perhaps due to the well-documented disparities in healthcare delivery (11). It is likely that these mutations are more common than recognized. Therefore, despite ethnicity, EOAD should be considered in all patients with typical symptoms.

Work-up for memory deficits should include ruling out other secondary causes, such as vasculitis, vitamin deficiencies, syphilis, thyroid disease, and cerebrovascular disease (i.e., multi-infarct dementia) as well as genetic testing on patients with clinically suspected early-onset dementia. Social history should include occupational exposures to blood or brain products, as well as an alcohol consumption history. Neuroimaging studies are appropriate, starting with MRI, but including PET CT when the diagnosis is in doubt. EOAD patients tend to have more severe atrophy consistent with the possibility of aggressive nature of the disease. Moreover, imaging findings of involvement of neocortical areas of parietal and occipital lobes (as compared to hippocampal areas in late onset Alzheimer's disease) in EOAD are consistent with clinical observations of language impairment (12). If a prion disease is entertained as a diagnosis, lumbar puncture should be performed with a modified Western blot on CSF for protein 14-3-3, followed by an EEG to detect the characteristic waveform of Creutzfeldt–Jacob disease.

Though it is estimated that 90% of EOAD genetic syndromes are autosomal recessive in nature, all three gene mutations associated with autosomal dominant EOAD have extremely high penetrance (Table 1) (6). Hence, genetic counseling should be offered to all family members of a patient diagnosed with a genetic form of dementia. EOAD patients progress more rapidly, have less clinical response to medications, and experience worse outcomes (12). Since little is known about best therapies in this population, early recognition is vital to the furthering of our understanding of the natural history and treatment of this condition. It is therefore imperative that patients are identified early in the course and enrolled in clinical trials as soon as possible.

**Table 1:** Review of genetics of early onset Alzheimer's dementia

Genes	Inheritance pattern	Chromosome	Penetrance
PSEN1	Autosomal dominant	14	Variable with up to 100%
PSEN2	Autosomal dominant	1	Variable with up to 100%
APP	Autosomal dominant	21	Variable with up to 100%
APP (673V)	Autosomal recessive	21	100% in homozygous state
